# PD 0332991对人内皮细胞增殖和凋亡的作用

**DOI:** 10.3779/j.issn.1009-3419.2018.05.04

**Published:** 2018-05-20

**Authors:** 辰龙 赵, 明辉 刘, 永文 李, 洪兵 张, 颖 李, 颢 宫, 茵 袁, 伟婷 李, 红雨 刘, 军 陈

**Affiliations:** 1 300052 天津，天津医科大学总医院肺部肿瘤外科 Department of Lung Cancer Surgery, Tianjin Medical University General Hospital, Tianjin 300052, China; 2 300052 天津，天津市肺癌研究所 Tianjin Key Laboratory of Lung Cancer Metastasis and Tumor Microenvironment, Tianjin Lung Cancer Institute, Tianjin Medical University General Hospital, Tianjin 300052, China

**Keywords:** 肺癌, 肿瘤血管生成, PD 0332991, Lung cancer, Tumor angiogenesis, PD 0332991

## Abstract

**背景与目的:**

肿瘤的血管生成（angiogenesis）是肿瘤发展中的一个重要过程。PD 0332991是一种细胞周期抑制剂，能特异性抑制CDK4/6的活性及细胞周期进展，在小鼠的移植瘤模型中，使用PD 0332991治疗的小鼠，其移植瘤中微血管生成明显减少、血管密度显著低于对照组，但其机理不明。本研究的目的在于探讨PD 0332991对血管内皮细胞的作用及分子机理。

**方法:**

采用EA.hy926血管内皮细胞作为研究模型。MTT法及EdU法检测PD 0332991对EA.hy926细胞活性和增殖能力的影响；划痕实验和transwell小室实验检测PD 0332991对内皮细胞迁移力的影响；流式细胞术检测PD 0332991对内皮细胞细胞周期及细胞凋亡的影响；Western blot检测PD 0332991对内皮细胞细胞周期相关蛋白表达的影响。

**结果:**

PD 0332991显著抑制EA.hy926内皮细胞的增殖、活性及迁移能力；使EA.hy926内皮细胞周期受阻滞并诱导其凋亡；抑制CDK4/6表达以及Rb的磷酸化，从而抑制内皮细胞细胞周期进展。

**结论:**

PD 0332991能抑制内皮细胞的增殖和活性，并诱导其凋亡。

## Abstract (en)

**Background and objective:**

Angiogenesis is an important process in the development of tumor. PD 0332991, a cell cycle inhibitor, can specifically inhibit CD4/6 phosphorylation and cell cycle progression. In xeongraft mice models, PD 0332991 treated mice had significantly decreased angiogenesis and vascular density compared with the control group, but the mechanism remains unknown. The purpose of this study is to investigate the role and molecular mechanism of PD 0332991 on vascular endothelial cells.

**Methods:**

EA.hy926 cells, a kind of vascular endothelial cell, were used as the research model. The effects of PD 0332991 on the activity and proliferation of EA.hy926 cells were detected by the MTT, EdU assays. Wound-healing assays and transwell assays were used to determine the effects of PD 0332991 on the mobility of EA.hy926. The influence of PD 0332991 on cell cycle and apoptosis of endothelial cells was tested by flow cytometry, and the Western blot was applied to observe the expression of cell cycle related proteins in EA.hy926 cells treated by PD 0332991.

**Results:**

PD 0332991 significantly inhibited the proliferation and mobility of EA.hy926 cells, caused cell cycle arrest and apoptosis. At the same time, PD 0332991 inhibited the expression of CDK4/6 and phosphorylation of Rb, and thus inhibited the cell cycle progression of EA.hy926 cells.

**Conclusion:**

PD 0332991 can inhibit the proliferation and activity of endothelial cells and induces apoptosis.

肺癌是呼吸系统最常见的恶性肿瘤，其发病率和死亡率在世界范围内居高不下，特别是在我国大中城市呈现逐年上升的趋势^[1-3]^。肺癌的发生发展是一个多步骤的复杂过程，涉及多个基因的异常调控^[4]^。近年来，随着肿瘤研究的深入，众多的证据^[5, 6]^显示，肿瘤的血管生成（angiogenesis）是肿瘤发展中的一个重要过程。肿瘤的生长有两个明显不同的阶段，即从无血管的缓慢生长阶段转变为有血管的快速增殖阶段，血管生成使肿瘤能够获得足够的营养物质，是促成生长速度转变的关键环节^[7]^。肿瘤的新生血管为不断侵袭生长的肿瘤提供氧气和营养，运走代谢废物，在原位肿瘤的形成和生长以及在转移瘤的形成中起着十分重要的作用。肿瘤血管生成是一个极其复杂的过程，一般包括血管内皮基质降解、内皮细胞移行、内皮细胞增殖、内皮细胞管道化分支形成血管环和形成新的基底膜等步骤^[8, 9]^。肿瘤血管生成的发生一方面是由于肿瘤细胞释放血管生成因子激活血管内皮细胞，促进内皮细胞的增殖和迁移，另外一方面肿瘤细胞也可以招募细胞外基质中的以及宿主细胞分泌的促血管生成因子，促进肿瘤血管生成^[5, 6]^。此外，VEGF/VEGFR信号通路系统作为一种内皮细胞的有丝分裂原和促血管生成因子，在原位肿瘤的形成和生长以及在转移瘤的形成中起着十分重要的作用^[7, 10, 11]^。

目前在肿瘤临床治疗中最常用的血管生成抑制剂为贝伐珠单抗，其为第一个联合化疗被用于晚期非小细胞肺癌的血管生成抑制剂^[12]^。ECOG4599临床实验的结果确定了贝伐珠单抗联合铂类、紫杉醇类化疗药物可用于晚期非鳞的非小细胞肺癌的一线治疗^[13]^。然而关于其他的血管生成抑制剂包括：舒尼替尼、索拉非尼和凡德他尼的临床实验中均未取得理想的实验结果^[10, 14]^。我们课题组在裸鼠的移植瘤动物模型中发现，使用细胞周期抑制剂PD 0332991后，小鼠的移植瘤中微血管生成明显减少、血管密度显著低于对照组^[15]^，本研究将深入探讨PD 0332991对人内皮细胞的作用，探讨PD 0332991通过抑制肺癌微血管形成抑制肺癌进展的作用及分子机理。

## 1 材料与方法

### 1.1 细胞株及主要仪器

EA.hy926人脐静脉细胞融合细胞，购自中国科学院细胞库，DMEM培养基、胎牛血清购自Life Technologies公司（Carlsbad, CA, USA）Palbociclib（PD 0332991）HCl购自Selleck，MTT（3-（4, 5-二甲基噻唑-2）-2, 5-二苯基四氮唑溴盐）试剂购自北京鼎国公司，EdU试剂盒购自锐博生物公司，细胞周期及凋亡试剂盒购自BD公司，免疫组化试剂盒、CD34抗体购自北京中杉金桥公司，VEGFR抗体购自Abcam公司，RB抗体，CDK4抗体，E2F1抗体、P-RBser795及P-RBser780抗体均来自Cell Signaling Technology公司（Danvers, MA, USA），CDK6抗体购自Sigma公司（Kansas, Missouri, USA），双报告酶标仪为tristar LB-941，流式细胞仪购自Beckman coulter公司（CA, USA）。

### 1.2 细胞培养及药物处理

EA.hy926细胞用含10%胎牛血清（fetal bovine serum, FBS）的DMEM（dulbecco′s modified eagle medium）培养，细胞培养于10 cm培养皿，置于37 ℃、5%CO_2_饱和湿度的培养箱中，0.25%胰酶-EDTA（ethylenediaminetetracetic acid）消化传代，所有实验均采用对数生长期细胞。Palbociclib（PD-0332991）HCl溶于于二甲基亚砜（dimethyl sulphoxide, DMSO）溶液中，药物处理浓度为IC_50_浓度或2倍、1/2倍IC_50_的浓度（根据实验不同）处理时间为48 h。

### 1.3 MTT分析PD 0332991对内皮细胞活性的影响

取对数期生长细胞，按每孔3×10^4^细胞铺于96孔板上，过夜待细胞贴壁后，设置分组，除药物浓度梯度组外，分设control组（只含细胞和培养基）和调零孔组（仅含培养基），PD 0332991药物各组分别按浓度梯度0、0.5 μmol/L、1 μmol/L、2 μmol/L、4 μmol/L、8 μmol/L、16 μmol/L、32 μmol/L、64 μmol/L递增，每组设6个复孔，每孔液体体积200 µL，于孵育箱分别孵育24 h、48 h、72 h，后每孔加入5 mg/mL的MTT 20 μL继续培养4 h，小心吸出培养液，每孔加入150 μL DMSO，置摇床上低速振荡10 min，使得结晶物充分溶解，用酶标仪测各孔的吸光值（490 nm）。计算时每组去掉最大值最小值，细胞抑制率＝（对照组吸光值-实验组吸光值）/对照组吸光值×100%，最终得出PD 0332991作用于EA.hy926 24 h/48 h/72 h的IC_50_浓度，实验独立重复3次。

### 1.4 EdU检测PD 0332991对内皮细胞DNA复制的影响

EdU（5-Ethynyl-2′-deoxyuridine）是一种胸腺嘧啶核苷类似物，能使细胞在DNA复制时，代替胸腺嘧啶（T）渗入正在合成的DNA分子中，基于Apollo^®^荧光染料与EdU的特异性反应即检测出DNA复制活性。细胞按每孔1×10^4^个铺于96孔板上，设置2组，分别为NC组和PD 0332991=4 μmol/L，每组处理3个复孔，其中，NC组只含培养基，PD 0332991组药物浓度为4 μmol/L，处理48 h后取出，弃掉培养基，PBS洗1次，5 min，后按Edu试剂盒说明书步骤进行处理。

### 1.5 划痕及迁移实验检测PD 0332991对内皮细胞迁移力的作用

划痕实验：取对数生长的EA.hy926细胞铺在6孔板上，设置3个组，分别为NC组、PD 0332991=6 μmol/L和PD 0332991=12 μmol/L，每组3个复孔，直至细胞生长到90%的密度，过夜饥饿处理，第2天用无菌的枪头划十字，并轻轻洗掉悬浮细胞，后分别加入2 mL含培养基（5%血清）、含PD 0332991 6 µmol/L、12 µmol/L的培养基，于显微镜下随时间变化观察拍照。

迁移实验：提前用PD 0332991药物处理处于对数生长期的EA.hy926细胞48 h，药物浓度分别为6 µmol/L、12 µmol/L，同时设置空白对照组。transwell小室中将提前处理好的3组细胞（NC、PD=6 μmol/L、PD=12 μmol/L）按密度1×10^5^个/孔铺于上室上，下室加入600 μL完全培养基，置于37 ℃、5%CO_2_恒温孵箱培养4 h。取出transwell小室用棉签小心拭去上室细胞，4%多聚甲醛固定10 min，结晶紫室温染色25 min，PBS洗3遍，显微镜下观察下室杯底，选5个随机视野进行拍照计数。计算平均每个视野的细胞数，以穿过滤膜进入下室杯底的细胞数来表示细胞的迁移能力。每组实验重复3次。

### 1.6 流式细胞仪检测PD 0332991对内皮细胞周期及凋亡的作用

#### 1.6.1 流式细胞仪检测PD 0332991对内皮细胞细胞周期的影响

取对数期生长的细胞，按每孔1.5×10^6^个铺于6孔板上，分设3组，分别为NC组、PD 0332991=2 μmol/L和PD 0332991=4 μmol/L，每组3个复孔，NC组为空白对照，只含培养基，处理细胞48 h，收集细胞，4 ℃预冷的1×PBS洗涤收集的细胞2次，1, 500 rpm-2, 000 rpm离心5 min，弃上清；少量4 ℃预冷1×PBS重悬细胞至单细胞悬液，逐滴加入4 ℃预冷的乙醇中（乙醇终浓度为50%-70%），吹打均匀，4 ℃透化过夜；上机前准备：6, 000 rpm离心透化后的细胞样品5 min，弃上清，预冷的PBS洗涤2次以充分去除残留的乙醇；500 μL周期染料（BD的PI周期试剂盒-550825）常温染色15 min-20 min；之后上机检测。

#### 1.6.2 流式细胞仪检测PD 0332991诱导内皮细胞凋亡的作用

取对数期生长的细胞，按每孔1.5×10^6^个铺于6孔板上，设3组，分别为PD 0332991=2 μmol/L、PD 0332991=4 μmol/L以及空白对照组，处理48 h后正常收集细胞；4 ℃预冷的1×Buffer洗涤收集的细胞2次，1, 000 rpm-1, 500 rpm离心5 min，弃上清；300 μL 4 ℃预冷1×Buffer重悬细胞至单细胞悬液，加入5 μL Annexin V-FITC，37 ℃孵育20 min，之后加入5 μL PI，吹打均匀，常温继续孵育10 min之后上机检测。

### 1.7 免疫组化（IHC）

石蜡包埋的小鼠移植瘤组织见课题组已发表的文献报道^[15]^。将石蜡包埋组织以5 µM连续切片展开，烘干，烤片，二甲苯脱蜡，梯度酒精脱水，阻断灭活内源性过氧化物酶，抗原修复：置0.01 M枸橼酸缓冲液（PH6.0）中用煮沸，自然冷却，正常羊血清工作液封闭，滴加一抗4 ℃孵育过夜，滴加生物素标记二抗37 ℃ 30 min，滴加辣根过氧化物酶标记的链霉素卵白素工作液，37 ℃孵育30 min，PBS冲洗3×5 min。DAB/H_2_O_2_反应染色，自来水充分冲洗后，苏木素复染，常规脱水，透明，干燥，封片。同时设置空白阴性对照，之后计数观察。

### 1.8 蛋白质免疫印记法（WB）

RIPA裂解蛋白，蛋白来自PD 0332991=4 μmol/L、PD 0332991=8 μmol/L处理48 h后的内皮细胞，同时设置无药物处理的空白对照组，于冰上裂解30 min，BCA法测蛋白浓度，加入5×SDS-PAGE蛋白缓冲液，充分混匀，100 ℃金属浴加热变性，取30 μg蛋白上样，80 V电泳2 h，100 V转膜65 min，含5%脱脂牛奶的1×TBST室温下，脱色摇床上封闭1 h。加入CDK4/6、RB、P-RBser^780^、P-RBser^795^、E2F1、VEGFR抗体，4 ℃孵育过夜，次日用TBST在室温下脱色摇床上漂洗3次，每次10 min，加HRP标记的二抗，室温孵育1 h，显色曝光。

### 1.9 统计学方法

应用graphpad prism 6.02软件统计分析作图，实验中组间比较采用两样本均数*t*检验，*P* < 0.05为差异有统计学意义。流式分析软件采用wincycle 6-16-03-F32。划痕与Western blot结果分析采用Image J 1.46r软件。

## 2 结果

### 2.1 PD 0332991抑制内皮细胞生长及增殖

MTT法检测细胞活性，结果显示EA.hy926经PD 0332991处理24 h、48 h与72 h的IC_50_分别为（10.279±2.971）µmol/L、（4.241±0.292）µmol/L及（2.574±0.692）µmol/L。EA.hy926细胞活性随药物浓度与时间梯度增加而降低。EdU染色以检测PD 0332991对内皮细胞的增殖的影响，结果显示，PD 0332991处理组（PD）阳性细胞数为（2.02±1.93），明显低于对照组（NC）（77.06±9.19，*P* < 0.000, 2），提示PD 0332991能够明显抑制EA.hy926细胞的增殖（图 1）。

**1:**
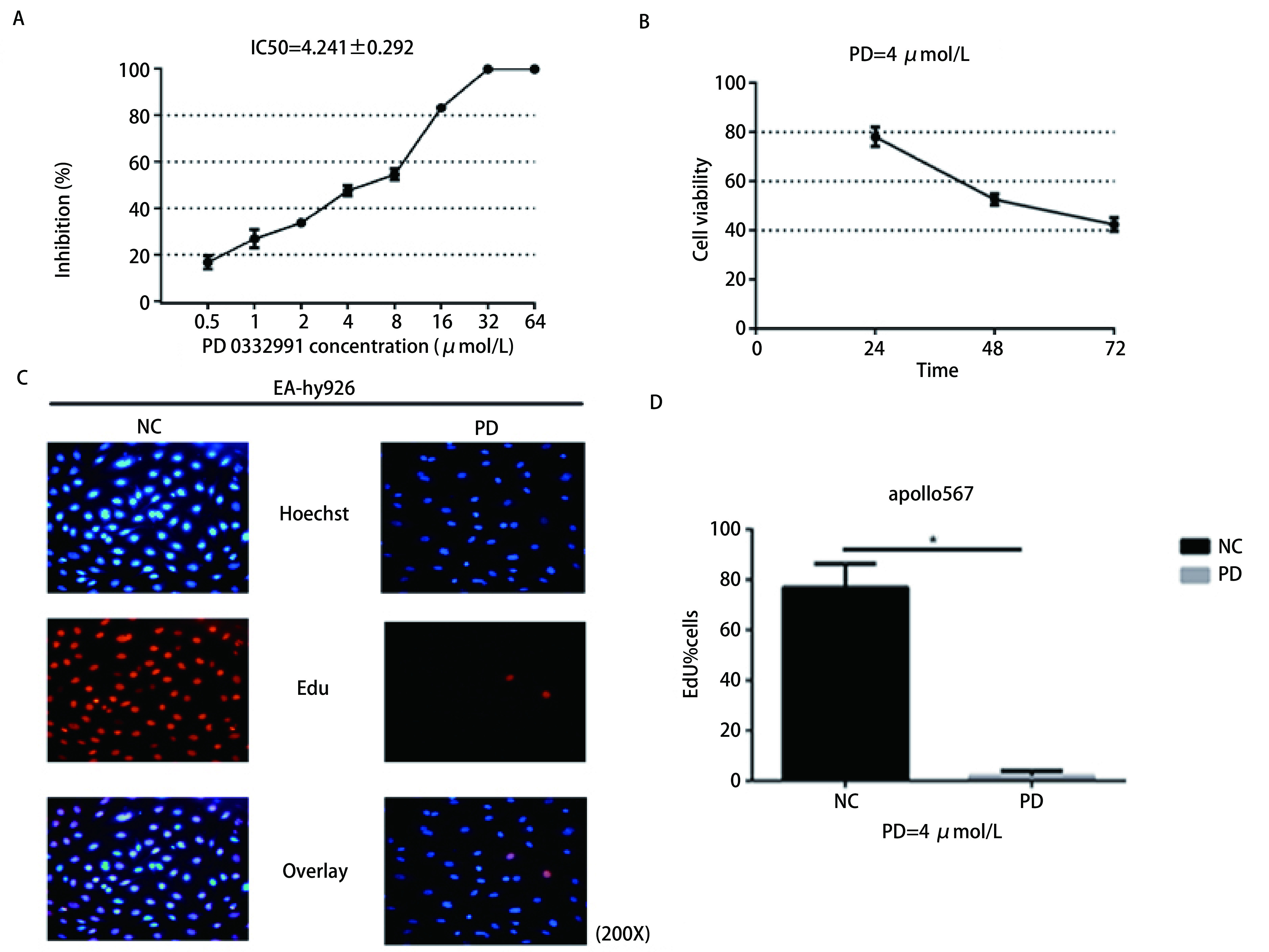
PD 0332991抑制内皮细胞的活性及增殖。A、B：PD 0332991处理内皮细胞后，细胞活性随药物浓度梯度和时间梯度降低；C、D：PD 0332991抑制内皮细胞增殖。 PD 0332991 inhibited the viability and proliferation of EA.hy926 cells. A, B: After PD 0332991 treatment, cell viability was reduced with the increase of drug concentration and time gradient; C, D: PD 0332991 inhibits the proliferation of EA.hy926 cells.

### 2.2 PD 0332991抑制内皮细胞的迁移

分别通过transwell法及划痕实验检测细胞的迁移能力，PD 0332991处理EA.hy926细胞后，与对照组相比较，处理组中穿过膜的细胞数量明显减少（图 2）。进一步应用细胞划痕实验证实，不同浓度的PD 0332991（6 µmol/L和12 µmol/L）处理6 h、18 h、32 h后，PD 0332991处理组的EA.hy926细胞迁移率与对照组相比明显下降（*P* < 0.05），而且随着PD 0332991药物浓度的增加，细胞迁移率下降趋势更明显（图 2）。

**2:**
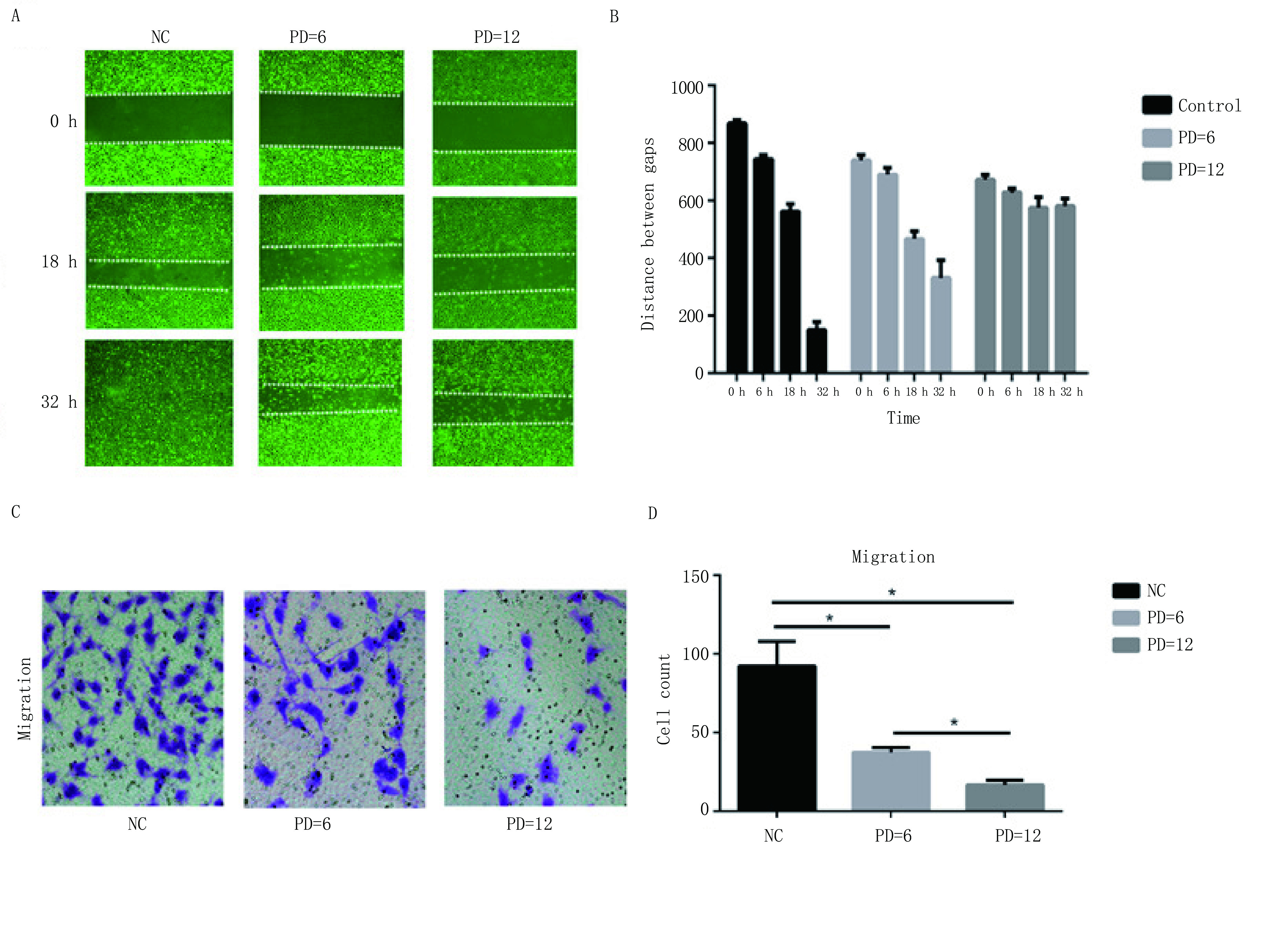
PD 0332991抑制内皮细胞的迁移。A、B：划痕实验检测PD 0332991对内皮细胞迁移能力的影响；C、D：Transwell实验检测PD 0332991对内皮细胞迁移能力的影响。 PD 0332991 inhibited the migration of EA.hy926 cells. A, B: The effect of PD 0332991 on endothelial cell migration was detected by wound-healing assay; C, D: Transwell migration assay was used to detect the effect of PD 0332991 on endothelial cell migration.

### 2.3 PD 0332991诱导细胞阻滞于G_1_期并诱导细胞凋亡

#### 2.3.1 PD0332991诱导细胞凋亡

用PD 0332991=4 µmol/L处理细胞48 h后，与对照组相比，处理组总的凋亡细胞明显增加，对照组与处理组凋亡细胞占总的细胞比例分别为（2.79±0.1）与（20.32±1.23，*P* < 0.001）；PD 0332991处理组早期凋亡及晚期凋亡细胞占比分别为（1.95±0.63、8.37±0.59），NC组早期凋亡及晚期凋亡细胞占比分别为（1.89±0.07、0.89±0.03），结果显示与对照组对比，处理组早期凋亡及晚期凋亡率均明显增加（*P* < 0.001）。表明PD 0332991能诱导EA.hy926细胞产生凋亡（图 3）。

**3:**
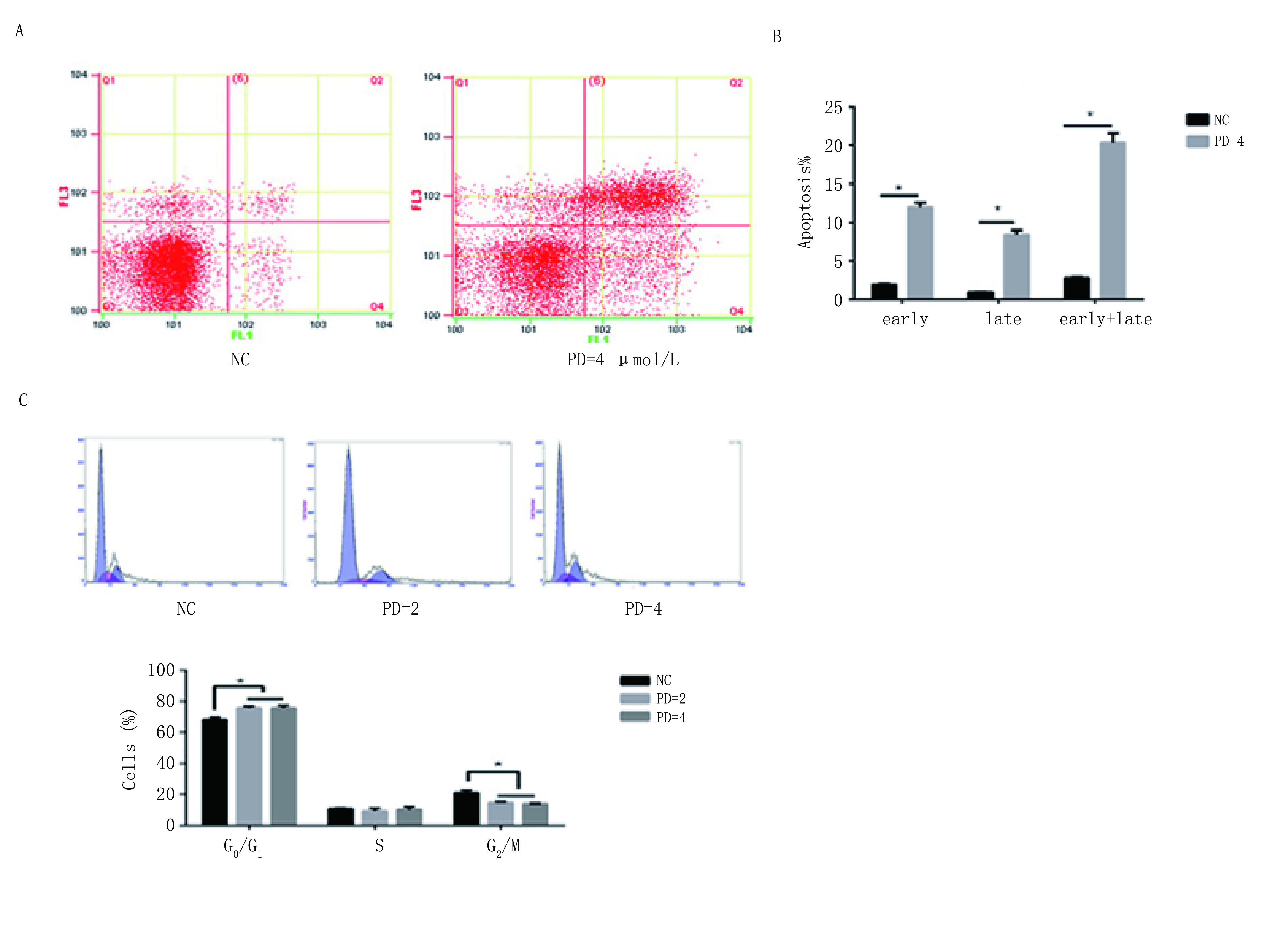
PD 0332991影响内皮细胞的细胞周期和凋亡。A-C：应用流式细胞技术检测发现PD 0332991处理EA.hy926 cells后，G_1_期细胞明显增多，细胞凋亡明显增加。 The effects of PD 0332991 on cell cycle and apoptosis. A-C: After treatment with PD 0332991 in EA.hy926 cells, we used flow cytometry to analyze the cell cycle and apoptosis.

#### 2.3.2 PD 0332991诱导细胞阻滞于G_1_期

NC组、PD=2 µmol/L、PD=4 µmol/L组，G_0_/G_1_期细胞比例分别为（68.1±1.81）、（75.8±1.2）与（75.73±1.7），NC组*vs* PD 0332991=2 µmol/L组（*P* < 0.05），NC组*vs* PD 0332991=4 µmol/L组（*P* < 0.05）；G_2_/M期细胞占比分别为（21.06±1.7）、（14.8±0.69）与（14±0.26），NC组*vs* PD 0332991=2 µmol/L组（*P* < 0.05），NC组*vs* PD 0332991=4 µmol/L组（*P* < 0.05）。数据显示PD 0332991处理后，G_0_期/G_1_期细胞比例增多，G_2_期/M期细胞比例减少，提示PD0332991诱导细胞阻滞于G_1_期，并减少处于G_2_期/M期的细胞。差异具有统计学意义（图 3）。

### 2.4 PD 0332991抑制移植瘤内血管新生

肺癌小鼠移植瘤的CD34免疫组化染色显示，PD 0332991处理组小鼠的移植瘤瘤体内CD34染色密度明显低于对照组，NC组与PD 0332991处理组MVD（肿瘤微血管密度）分别为（18.75±2.22）与（13±4, *P*=0.038）（图 4）。

**4:**
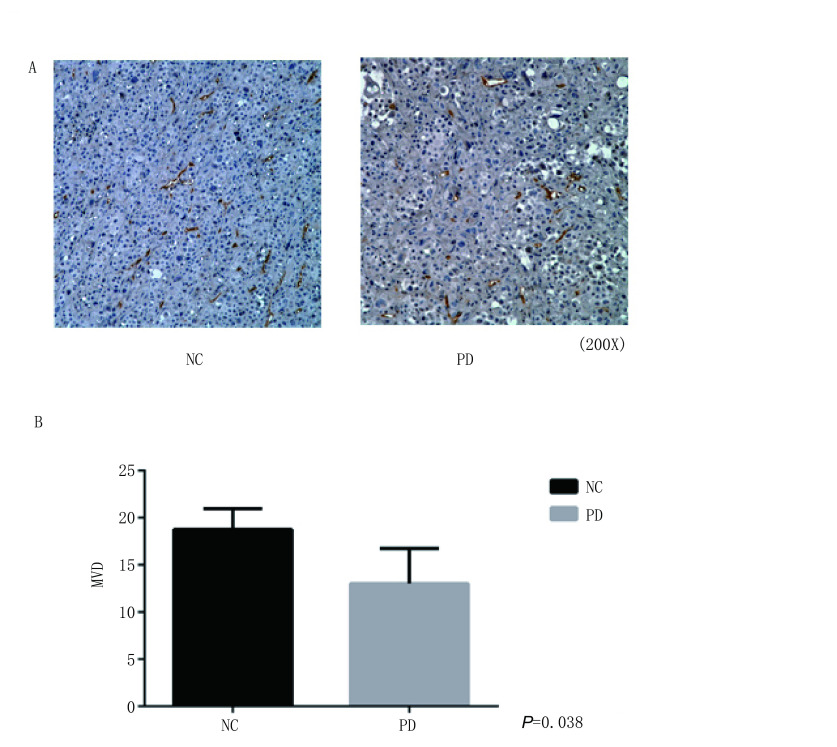
PD 0332991处理后的小鼠肿瘤血管密度分析。在PD 0332991处理的小鼠肺癌动物模型试验中，应用CD34抗体检测发现肿瘤模型中的血管密度明显降低。 Analysis of tumor vascular density in xeongraft mice model treated with PD 0332991. In the mouse model of lung cancer treated with PD 0332991, CD34 antibody detection revealed a marked decrease of vascular density in the tumor tissues.

### 2.5 PD 0332991降低细胞周期相关蛋白的表达

经PD 0332991处理的EA.hy926内皮细胞，P-RBser^795^及P-RBser^780^的水平明显降低，同时细胞周期相关蛋白CDK6、CDK4和E2F1表达明显减少（图 5），提示PD 0332991通过抑制细胞周期相关蛋白而抑制内皮细胞细胞周期进展。

**5:**
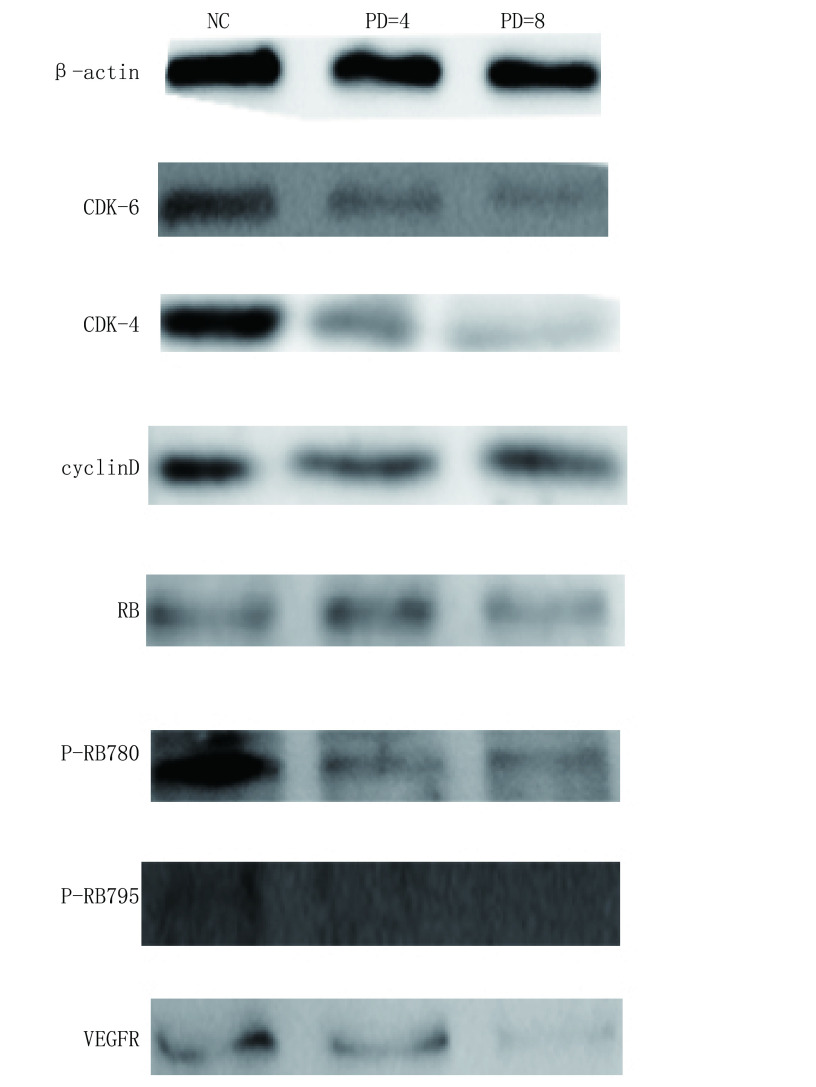
PD 0332991降低细胞周期相关蛋白的表达。在EA.hy926内皮细胞中经过PD 0332991处理后，应用Western blot检测发现与细胞周期相关诸多蛋白（如CDK6、CDK4、E2F1等）的表达水平发生了明显改变。 PD 0332991 reduces cell cycle-related protein expression. After treatment with PD 0332991 in endothelial cells, the expression levels of many proteins associated with the cell cycle (such as CDK6, CDK4, E2F1, etc.) were significantly changed by Western blot.

## 3 讨论

PD 0332991最早于2001年合成，为低毒的口服剂，能高选择性地结合于CDK4和CDK6 ATP结合口袋，抑制cyclinD-CDK复合物的活性，因此抑制Rb蛋白的磷酸化而导致细胞周期的阻滞^[16, 17]^。但在早期的临床试验中，PD 0332991对肿瘤的治疗作用一直存在很多的争议和不确定性，使其自问世以来，受到的关注并不多。2009年，Finn等^[16]^将PD 0332991用于一系列的乳腺癌细胞株的体外实验中发现，ER阳性的乳腺癌细胞株对PD 0332991高度敏感，之后，他们将PD 0332991与来曲唑合用，在12例ER受体阳性的患者中，有3例患者的肿瘤达到消退30%的疗效^[18]^。而Ⅰ期/Ⅱ期临床试验显示，较单独使用来曲唑组，PD 0332991联合来曲唑组PFS显著延长（HR=0.488; 95%CI: 0.319-0.748）^[19]^。因此2015年2月，美国FDA批准PD 0332991联合来曲唑，用于绝经后ER（+）/Her2（-）的乳腺癌的治疗。随后的三期临床试验更是奠定了PD 0332991联合雌激素抑制剂在晚期乳腺癌患者治疗中的地位^[20, 21]^。

PD 0332991是细胞周期蛋白CDK4/6高度选择性的抑制剂，其发挥作用主要是通过抑制复合物CDK4/6-CyclinD1的形成，从而抑制Rb磷酸化使得与其结合的E2F1转录因子不能被释放参与到细胞周期G_1_-S期的调节，从而使得肿瘤细胞阻滞于G_1_期^[17, 22, 23]^。在一二期临床试验中单用PD0332991在小儿畸胎瘤，套淋巴细胞瘤，精原细胞瘤，乳腺癌，以及脂肪肉瘤中彰显出临床获益^[23, 24]^。PD 0332991联合用药更是展现出令人鼓舞的效果，其联合蛋白酶体选择性可逆抑制剂Bortezomib治疗骨髓瘤^[25]^，联合布鲁顿酪氨酸激酶抑制剂依鲁替尼或PI3K抑制剂治疗套细胞淋巴瘤^[26, 27]^，联合MEK或BRAF抑制剂治疗黑色素瘤和结直肠癌^[28]^。特别的在乳腺癌中PD0332991联合来曲唑，联合氟维司群相对单用雌激素抑制剂显著提高病人无进展生存期^[19, 20]^。本实验中在对PD 0332991在血管内皮细胞EA.hy926中的研究中发现，其可以通过抑制细胞周期于G_1_期从而促进血管内皮细胞的凋亡，抑制其增殖和迁移，进而抑制血管的生成，从而抑制肿瘤的生长。
